# Transcription factor RUNX2 up-regulates chemokine receptor CXCR4 to promote invasive and metastatic potentials of human gastric cancer

**DOI:** 10.18632/oncotarget.8236

**Published:** 2016-03-21

**Authors:** Zheng-Jun Guo, Lang Yang, Feng Qian, Yan-Xia Wang, Xi Yu, Cheng-Dong Ji, Wei Cui, Dong-Fang Xiang, Xia Zhang, Peng Zhang, Ji Ming Wang, You-Hong Cui, Xiu-Wu Bian

**Affiliations:** ^1^ Institute of Pathology and Southwest Cancer Center, and Key Laboratory of Tumor Immunopathology, Ministry of Education of China, Third Military Medical University, Chongqing, China; ^2^ Department of General Surgery, Southwest Hospital, Third Military Medical University, Chongqing, China; ^3^ Laboratory of Molecular Immunoregulation, Cancer and Inflammation Program, Center for Cancer Research, National Cancer Institute, Frederick, MD, USA; ^4^ Collaborative Innovation Center for Cancer Medicine, Sun Yat-sen University, Guangzhou, China

**Keywords:** RUNX2, gastric cancer, invasion, metastasis, CXCR4

## Abstract

Runt-related transcription factor 2 (RUNX2) is a regulator of embryogenesis and development, but has also been implicated in the progression of certain human cancer. This study aimed to elucidate the role of RUNX2 in the invasive and metastatic potentials of human gastric cancer (GC) and the underlying mechanisms. We found that the levels of RUNX2 expression in gastric cancer tissues were correlated with the differentiation degrees, invasion depth and lymph node metastasis. COX regression analysis indicated that RUNX2 was an independent prognostic indicator for GC patients. RUNX2 significantly increased the migration and invasion ability of GC cells *in vitro* and enhanced the invasion and metastatic potential of GC cells in an orthotopic GC model of nude mice. Mechanistically, RUNX2 directly bound to the promoter region of the gene coding for the chemokine receptor CXCR4 to enhance its transcription. CXCR4 knockdown or treatment with AMD3100, a CXCR4 inhibitor, attenuated RUNX2-promoted invasion and metastasis. These results demonstrate that RUNX2 promotes the invasion and metastasis of human GC by transcriptionally up-regulating the chemokine receptor CXCR4. Therefore, the RUNX2-CXCR4 axis is a potential therapeutic target for GC.

## INTRODUCTION

Gastric cancer (GC) is one of the most lethal malignancies with distinctly high incidence and mortality in Asian countries [1]. Despite advances in diagnosis and treatment, the 5-year overall survival of GC patients remains at approximately 28% [2], mainly due to advanced stage of disease at diagnosis and limited understanding of the molecular mechanisms that control GC progression.

Recently, the involvement of Runt-related transcription factor 2 (RUNX2) in cancer development has been increasingly recognized. RUNX2 was initially identified as an osteoblast-specific transcription factor and a promoter of osteoblast differentiation [3]. RUNX2 deficient mice died shortly after birth with skeletal abnormalities of cleidocranial dysplasia (CCD) [4]. Osteoblast maturation was controlled by RUNX2 via target genes which facilitate bone development [5]. In breast cancer, RUNX2 mediated the interaction of cancer cells with bone microenvironment and promoted osteolytic destruction, partly by up-regulating the invasion-related gene MMP13 [6, 7]. In prostate cancer, RUNX2 was associated with skeletal destruction by enhancing the expression of metastasis-related proteins MMP9 and MMP13 [8]. Nevertheless, little is known about the role of RUNX2 in the progression of GC. In this study, we demonstrate that the level of RUNX2 expression was correlated with the invasion and lymph node metastasis of human GC. High expression of RUNX2 in tumors was associated with poor outcome of GC patients. Mechanistically we demonstrate that RUNX2 directly bound to CXCR4 promoter and enhanced its expression to promote GC invasion and metastasis. Thus, RUNX2/CXCR4 axis acts as a prognostic indicator and potential therapeutic target for human GC.

## RESULTS

### RUNX2 is highly expressed in human GC tissues and predicts prognosis of patients

**Figure 1:**
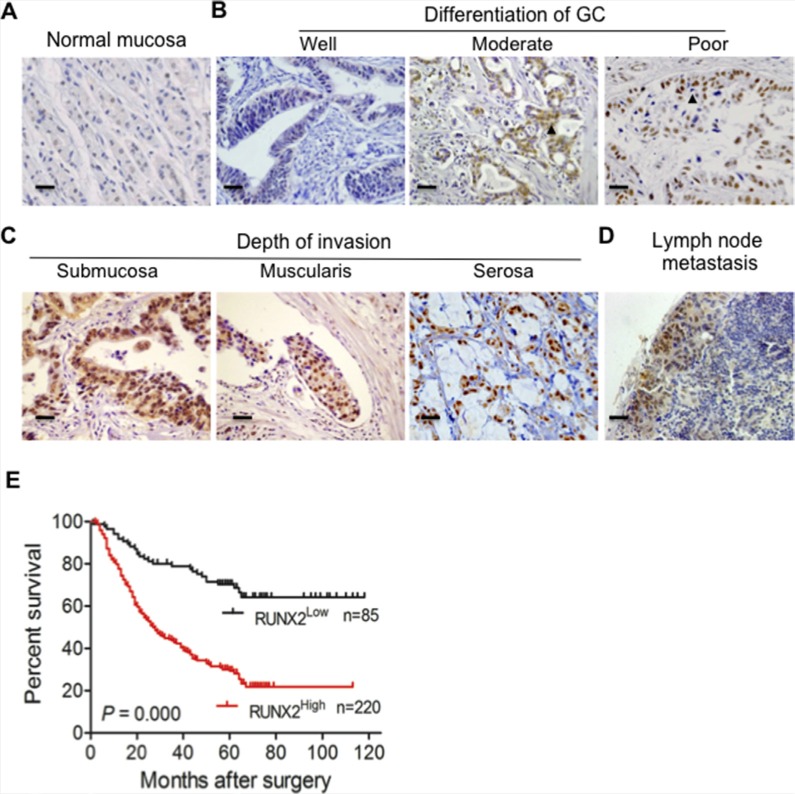
The expression of RUNX2 in human GC specimens is correlated with the outcome of GC patients (**A**) RUNX2 is not or only weakly expressed in normal gastric tissue as detected by IHC staining. (**B** and **C**) RUNX2 expression in GC tissues is correlated with different stages of differentiation and depth of tumor invasion. Arrows indicate RUNX2 positive GC cells. (**D**) Positive staining of RUNX2 in GC metastatic foci of lymph node. (**E**) Kaplan-Meier Overall survival curves indicate that patients with RUNX2^High^ staining have shorter life time after surgery than patients with RUNX2^Low^ tumors (RUNX2^High^, *n* = 220 and RUNX2^Low^, *n* = 85). Scale bar = 50 μm.

To examine the relevance of RUNX2 expression to human GC progression, we first analyzed the expression of RUNX2 in 305 GC specimens and paired adjacent gastric tissues by immunohistochemisty (IHC). RUNX2 was not or only weakly expressed in normal gastric mucosa (Figure 1A). High-level expression of RUNX2 protein was detected in 220 of 305 (72.1%) GC tissues as compared to adjacent gastric tissues (66/305, 21.6%) (*P* < 0.01, Table 1). Analysis of the relationship between RUNX2 expression and clinicopathological features of GC showed that high expression of RUNX2 was correlated with low differentiation of human GC (*P* < 0.05, Figure 1B and Table 1). RUNX2 level was positively correlated tumor invasion depth (Figure 1C), lymph node metastasis (Figure 1D) and TNM status (*P* < 0.01 for all, Table 2). Kaplan-Meier (K-M) analysis showed that patients with high RUNX2 expression in tumors had a shorter lifespan than those with low RUNX2 expression in tumors (*P* < 0.01, Figure 1E). COX's proportion hazard regression analysis indicated that RUNX2 was an independent prognostic indicator of the outcome of GC patients (*P* < 0.01, Table S1). These results suggest that RUNX2 may serve as a prognostic predictor for GC patients.

**Table 1:** RUNX2 IHC staining in gastric cancer tissues and adjacent tissues

Tissue types	RUNX2 expression (*n* = 305)		*P* value
	Low (%)	High (%)	
Gastric cancer tissues	85 (27.9)	220 (72.1)	< 0.01
Adjacent tissues	239 (78.4)	66 (21.6)	

**Table 2:** The correlation between RUNX2 expression in tumors and clinicopathological features of GC patients

Feature	RUNX2 expression (*n* = 305)		
	Low (*n* = 85)	High (*n* = 220)	*P* values
Gender			
Male	60	146	0.480
Female	25	74	
Age			
< 60 years	51	119	0.352
≥ 60 years	34	101	
Differentiation degrees			
High	15	29	0.012
Moderate	17	20	
Low	53	171	
TNM Stages			
TNM 1 + 2	49	75	< 0.01
TNM 3 + 4	36	145	
Lymph node metastasis			
Positive	42	161	< 0.01
Negative	43	59	
Depth of invasion			
Submucosa & Muscularis	38	42	< 0.01
Serosa	47	178	

### RUNX2 promotes the migration and invasion of GC cells *in vitro*

**Figure 2:**
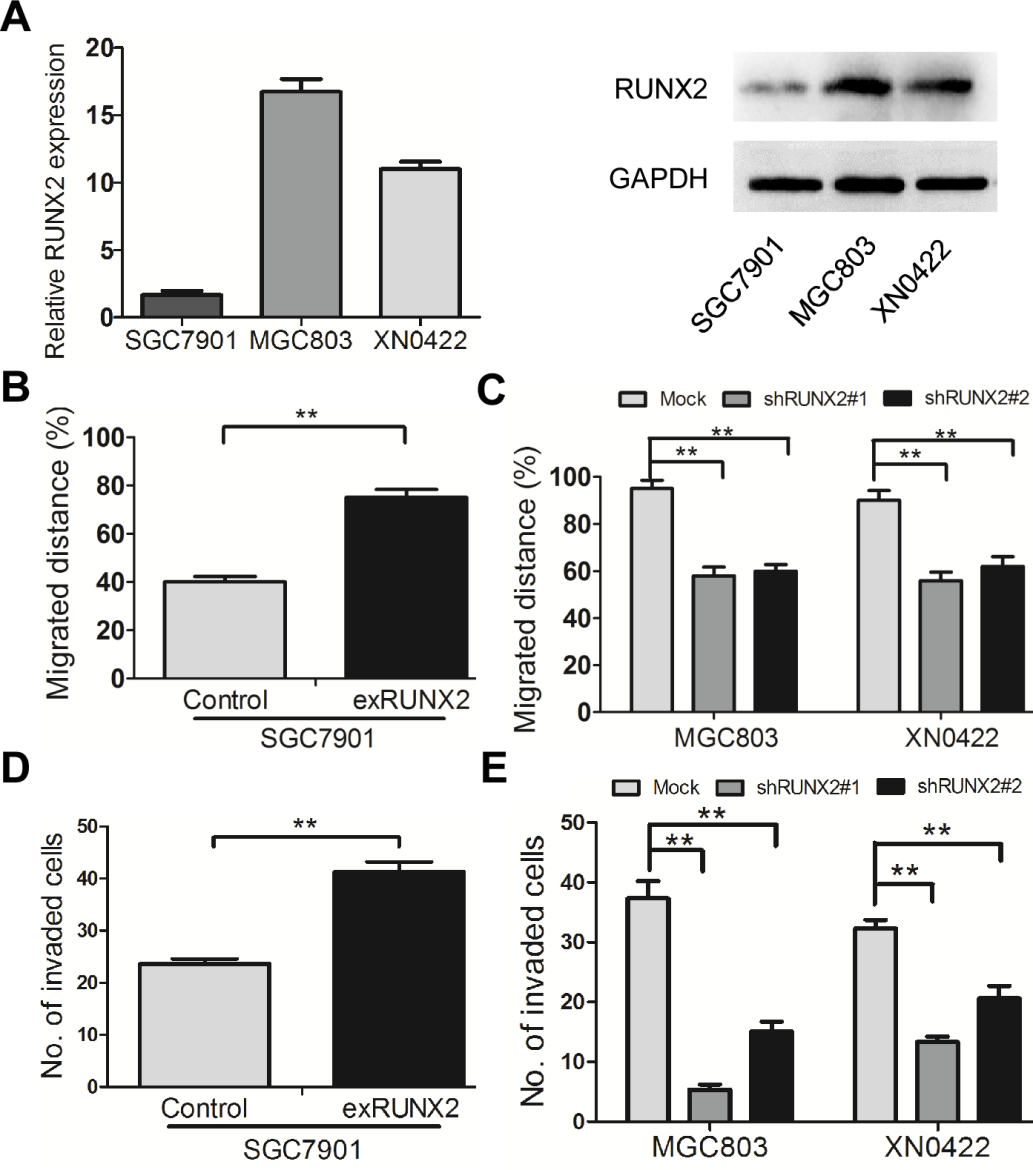
RUNX2 promotes the migration and invasion of GC cells *in vitro* (**A**) The expression level of RUNX2 in SGC7901 cells is lower than that in MGC803 and XN0422 cells at mRNA and protein levels detected by qPCR and WB. (**B**) The cell scratching assay shows that RUNX2-overexpressing SGC7901 cells migrate at longer distance than control cells. (**C**) After silencing RUNX2, the migrated distance of MGC803 and XN0422 cells was significantly shortened as compared with mock cells. (**D**) RUNX2-overexpressing SGC7901 cells showed higher invasiveness than control SGC7901 cells as analyzed in invasion assay. (**E**) RUNX2-knockdown in both MGC803 and XN0422 cells impaired their invasion ability. **P < 0.01, Student's *t* test.

RUNX2 was expressed at a low level in the human GC cell line SGC7901, as compared to MGC803 GC cells and XN0422 primary cells (Figure 2A). To investigate the role of RUNX2 in GC invasion and metastasis, RUNX2-overexpressing (exRUNX2) and knockdown cell lines (shRUNX2) were used, which were established from SGC7901 cells (RUNX2 low) and MGC803 cells as well as XN0422 primary GC cells (RUNX2 high), respectively (Supplementary Figure S1A–S1C). Cell monolayer scratching assays showed that the migration ability of SGC7901-exRUNX2 cells was significantly increased as compared to SGC7901-Control cells (Figure 2B and Supplementary Figure S2A). In contrast, MGC803-shRUNX2 and XN0422-shRUNX2 cells showed significantly decreased motility as compared to mock transfected cells (Figure 2C and Supplementary Figure S2B, S2C). Invasion assays with Matrigel Transwells showed that RUNX2 over-expression enhanced the invasiveness of SGC7901 cells (Figure 2D and Supplementary Figure S2D), whereas knockdown of RUNX2 potently reduced the invasiveness of MGC803 and XN0422 cells (Figure 2E and Supplementary Figure S2E, S2F). These results suggest that RUNX2 expression is associated with increased motility and invasiveness of GC cells *in vitro*.

### RUNX2 promotes the invasion and metastasis of GC in orthotopic mouse model

**Figure 3:**
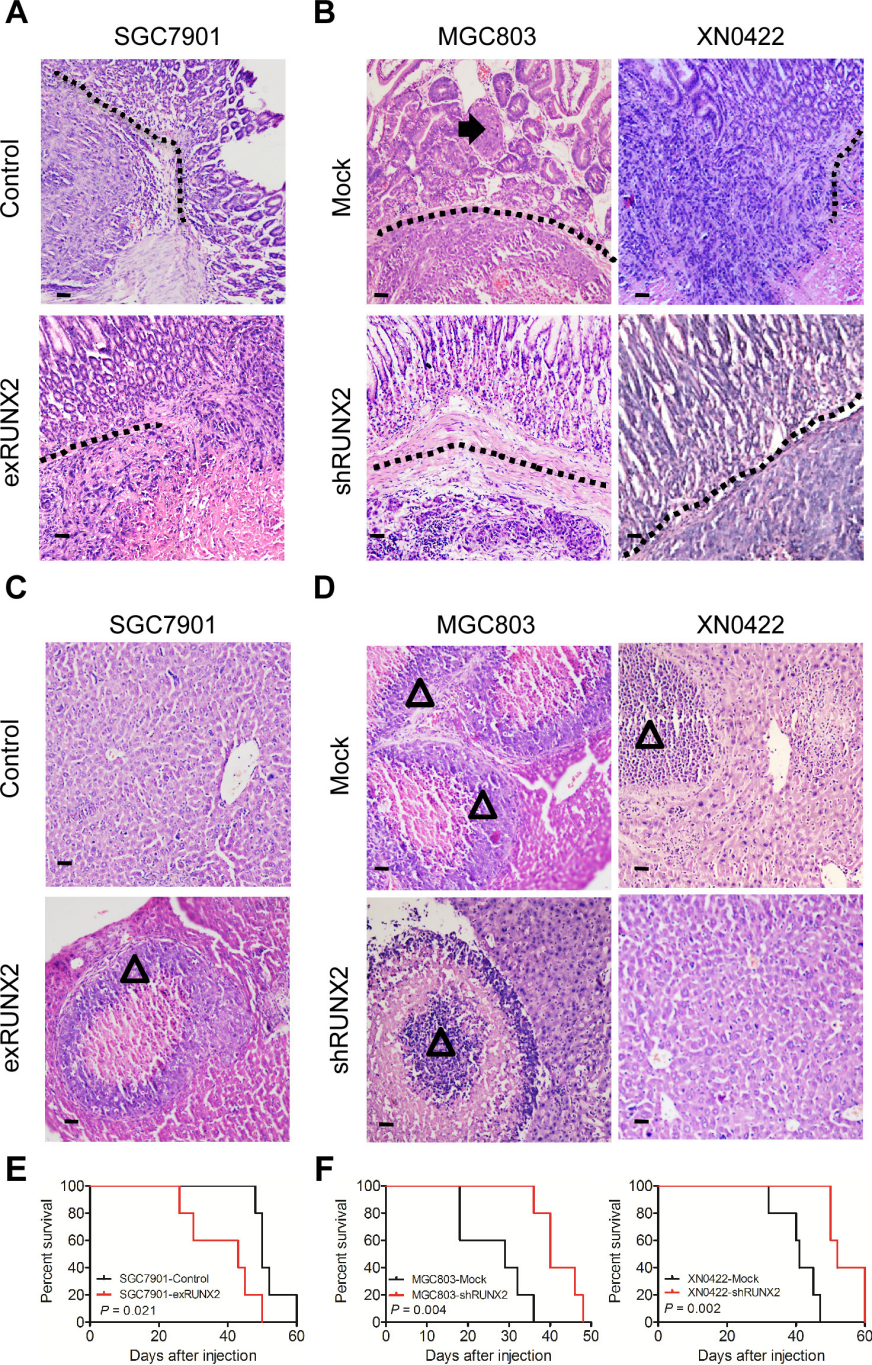
RUNX2 enhances GC cell invasion and metastasis in orthotopic transplantation mouse model (**A**) GC cells genetically engineered to overexpress or silence RUNX2 were implanted into the stomach serosa of nude mice (*n* = 5 for each group). The invasion and metastasis of transplanted tumors were examined after eight weeks. Representative images of orthotopic xenograft tumor sections show enhanced invasion abilitiy of tumors formed by RUNX2-overexpressing SGC7901 cells, as compared to SGC7901-Control cells. Black dotted line indicates the submucosa of the stomach. (**B**) Representative images show that RUNX2-knockdown in MGC803 and XN0422 cells impairs the invasiveness of xenografts. Black arrow shows tumor cell invasion into the mucosa. (**C**) Representative images showing liver metastasis of tumors formed by RUNX2-overexpressing SGC7901 cells as compared to SGC7901-Control cells. Empty triangle shows liver metastatic foci. (**D**) Representative images show that RUNX2-knockdown in MGC803 and XN0422 cells impairs their metastatic potential. Empty triangle shows liver metastatic foci. (**E**) Overall survival curves show that mice implanted with RUNX2-overexpressing SGC7901 cells have a shorter lifespan than mice implanted with control SGC7901 cells (*n* = 5 for each group). (**F**) Mice implanted with RUNX2-knockdown MGC803 cells and XN0422 primary cells show better outcome than their counterparts (*n* = 5 for each group).

We further examined the relationship of RUNX2 to the invasiveness and metastasis of human GC cells in a modified orthotopic tumor implantation model, in which genetically engineered GC cells were injected into the stomach subserosa of nude mice. Eight weeks after implantation, increased number of tumors infiltrating muscularis and mucosa were observed in the stomach of mice implanted with SGC7901-exRUNX2 cells as compared to control cells (*P* < 0.05; Figure 3A and Supplementary Table S2). Depletion of RUNX2 from MGC803 and XN0422 cells reduced tumor invasiveness (*P* < 0.01; Figure 3B and Supplementary Table S2). Metastatic foci in the liver were more frequently observed in mice injected with SGC7901-exRUNX2 cells as compared with mice injected with SGC7901-Control cells (*P* < 0.05; Figure 3C and Supplementary Table S2), while the frequency of metastasis was significantly lower in mice implanted with MGC803-shRUNX2 and XN0422-shRUNX2 cells as compared with mice implanted with mock cells (*P* < 0.01 and *P* < 0.05, respectively; Figure 3D and Supplementary Table S2). K-M survival curves indicated a shortened lifespan of mice implanted with SGC790-exRUNX2 cells (*P* < 0.05, Figure 3E). In contrast, a prolonged lifespan was observed in mice injected with MGC803-shRUNX2 and XN0422-shRUNX2 cells (*P* < 0.01, Figure 3F). Therefore, RUNX2 is closely related to the increased invasiveness and metastasis of GC cells *in vivo*.

### RUNX2 binds to CXCR4 promoter and up-regulates CXCR4 expression

**Figure 4:**
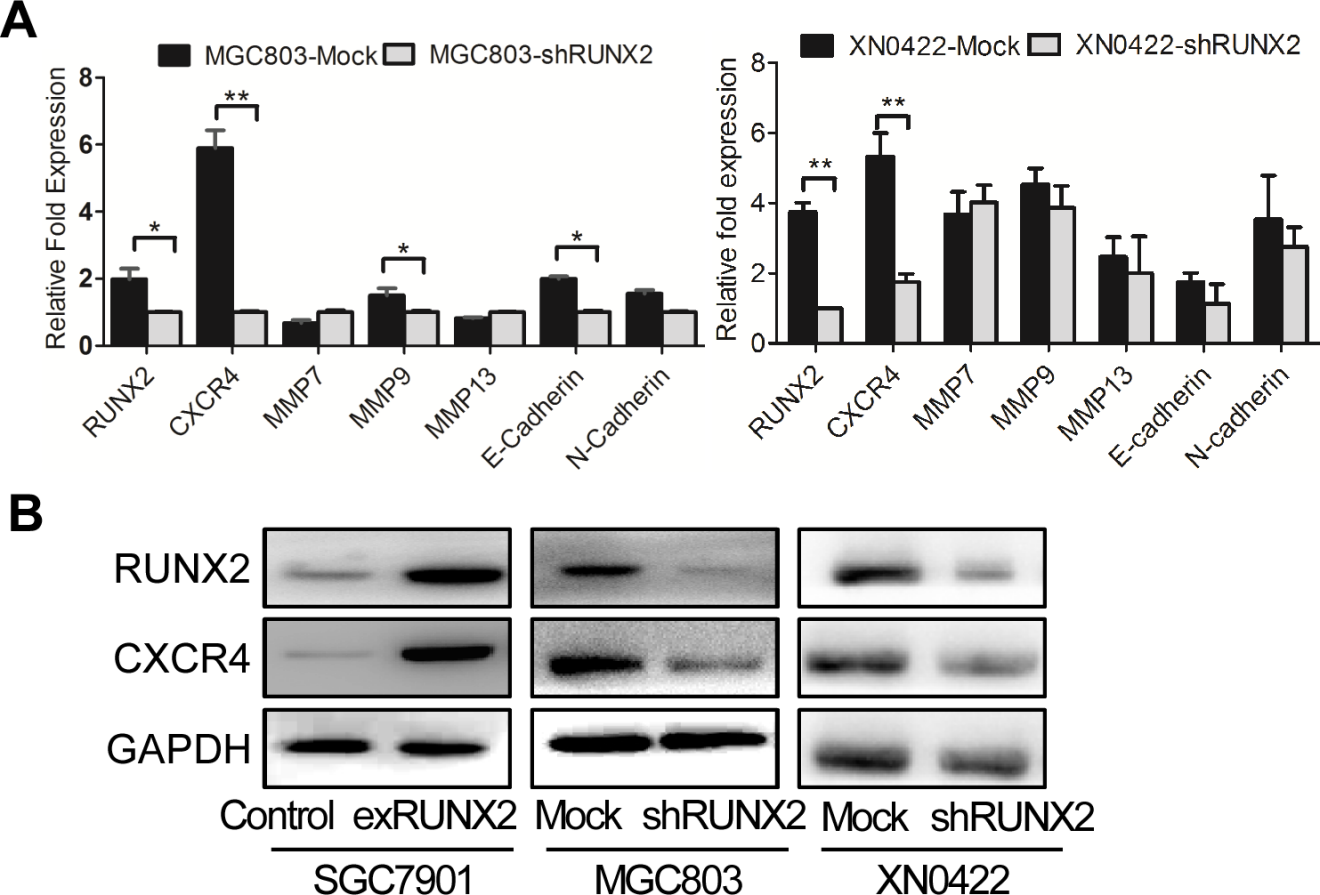
CXCR4 is a candidate RUNX2-targeting gene (**A**) Among invasion-related candidate RUNX2-targeting genes predicted by bioinformatic analysis, CXCR4 gene was markedly reduced by RUNX2 knockdown in both MGC803 cells (left) and XN0422 cells (right) as detected by qPCR. The expression level of genes was presented as relative fold increased normalized with GAPDH. *P < 0.05, **P < 0.01, Student's *t* test. (**B**) Western Blot shows increased CXCR4 in RUNX2-overexpressing SGC7901 cells but decreased CXCR4 in RUNX2-silenced MGC803 and XN0422 cells.

**Figure 5:**
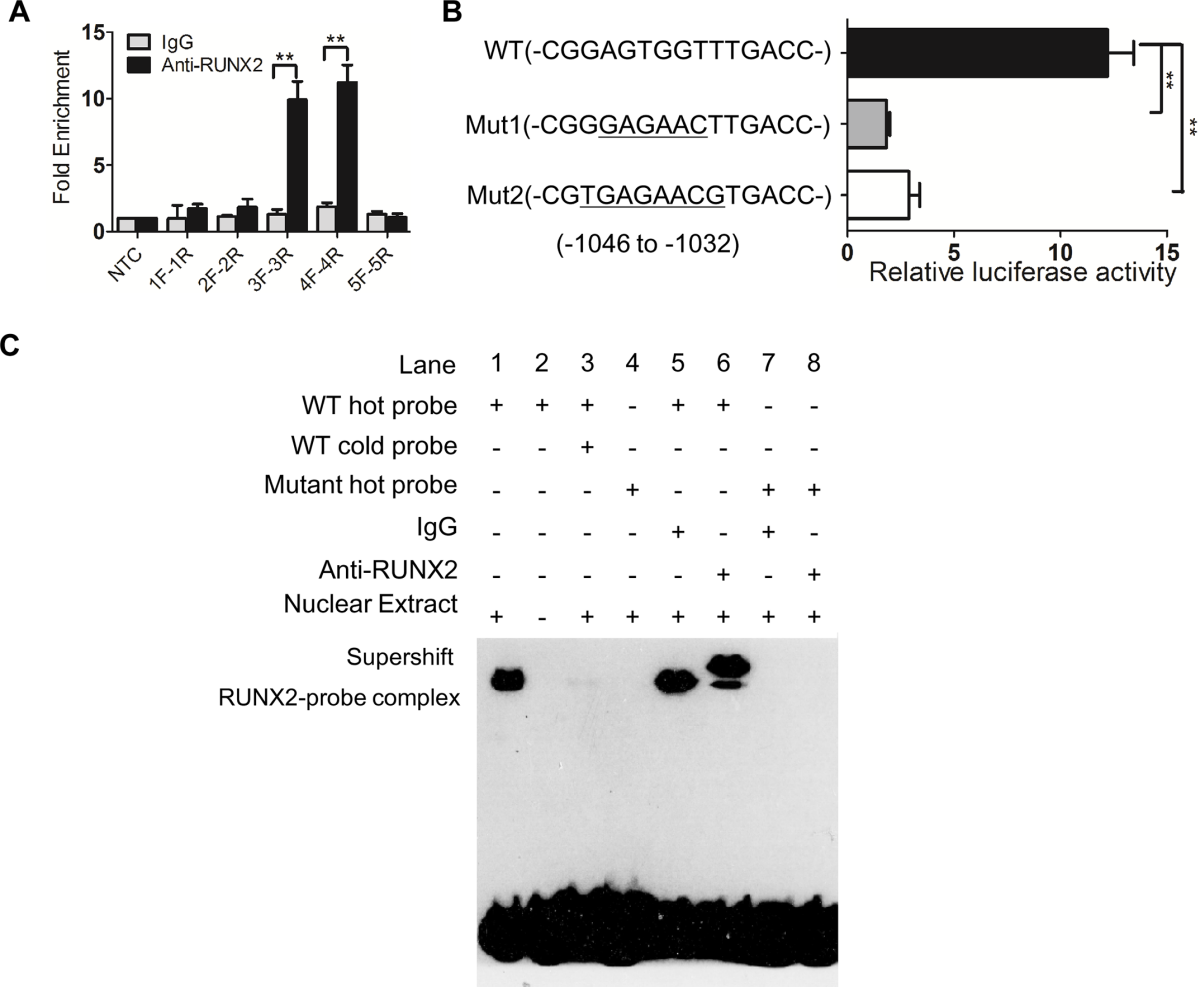
RUNX2 binds to with CXCR4 promoter (**A**) ChIP shows a candidate binding region (3F to 4R, 277 bp) of RUNX2 in CXCR4 promoter. (**B**) Luciferase activity assay shows that the predicted site (−1046 to −1032), but not mutants, is bound by RUNX2 in CXCR4 promoter. (**C**) Direct binding of RUNX2 to CXCR4 promoter region determined by EMSA. Lane 1 and 4 for nuclear extract binding reaction; lane 2 for negative control; lane 3 for competition test; lane 5 to 8 for supershift. **P < 0.01, Student's *t* test.

We next investigated the mechanistic basis for the capacity of RUNX2 to regulate the invasion and metastasis of GC cells. Since RUNX2 is a transcription factor, we used bioinformatics analysis to identify genes potentially targeted by RUNX2 [9] W. Among genes linked to tumor cell invasion and metastasis, six candidates were identified with the highest score for the gene encoding a chemokine receptor CXCR4 (Supplementary Table S3). In GC cell lines, with knockdown of RUNX2, the expression of CXCR4 mRNA was significantly down-regulated in both MGC803 and XN0422 cells (Figure 4A). The expression of CXCR4 protein was also significantly down-regulated by RUNX2 silencing in both MGC803 and XN0422 cells. Whereas, RUNX2 overexpression up-regulated CXCR4 in SGC7901 cells (Figure 4B). Further bioinformatics analysis revealed a putative RUNX2 binding site in the promoter of CXCR4 gene at −1046 to −1032 bp (CGGAGTGGTTTGACC). To examine the interaction between RUNX2 and the promoter of CXCR4, ChIP analysis was performed by using five pairs of primers (Supplementary Table S4) covering −2875 to +155 bp of the CXCR4 promoter. After immunoprecipitation, extracted DNA fragments amplified by qPCR (Figure 5A) indicated a putative 277 bp fragment in CXCR4 promoter (−1081 to −805) as a potential binding region for RUNX2, which contains the predictive binding site −1046 to −1032 in CXCR4 promoter.

Luciferase reporter assays further showed the predictive site bp −1046 to −1032 (CGGAGTGGTTTG ACC) in CXCR4 promoter bound by RUNX2 (Figure 5B), whereas the mutants (Mut1, CGGGAGAACTTGACC; Mut2, CGTGAGAACGTGACC) of bp −1046 to −1032 did not interact with RUNX2. The binding between RUNX2 and bp −1046 to −1032 in CXCR4 promoter was confirmed by using EMSA with labeled CGGAGTGGTTTGACC (WT) as a probe and nuclear extract from SGC7901-exRUNX2 cells (Figure 5C, lane 1). A supershift band was detected when an anti-RUNX2 antibody, but not control IgG, was incubated with the mixture of labeled WT probe (Figure 5C, lane 5 and 6), but not mutant (Mut1 as used) probe (Figure 5C, lane 7 and 8). These results indicate that RUNX2 directly binds to the promoter region of CXCR4 gene, resulting in its overexpression in human GC cells.

### CXCR4-mediated GC cell invasion and metastasis are associated with RUNX2 overexpression

**Figure 6:**
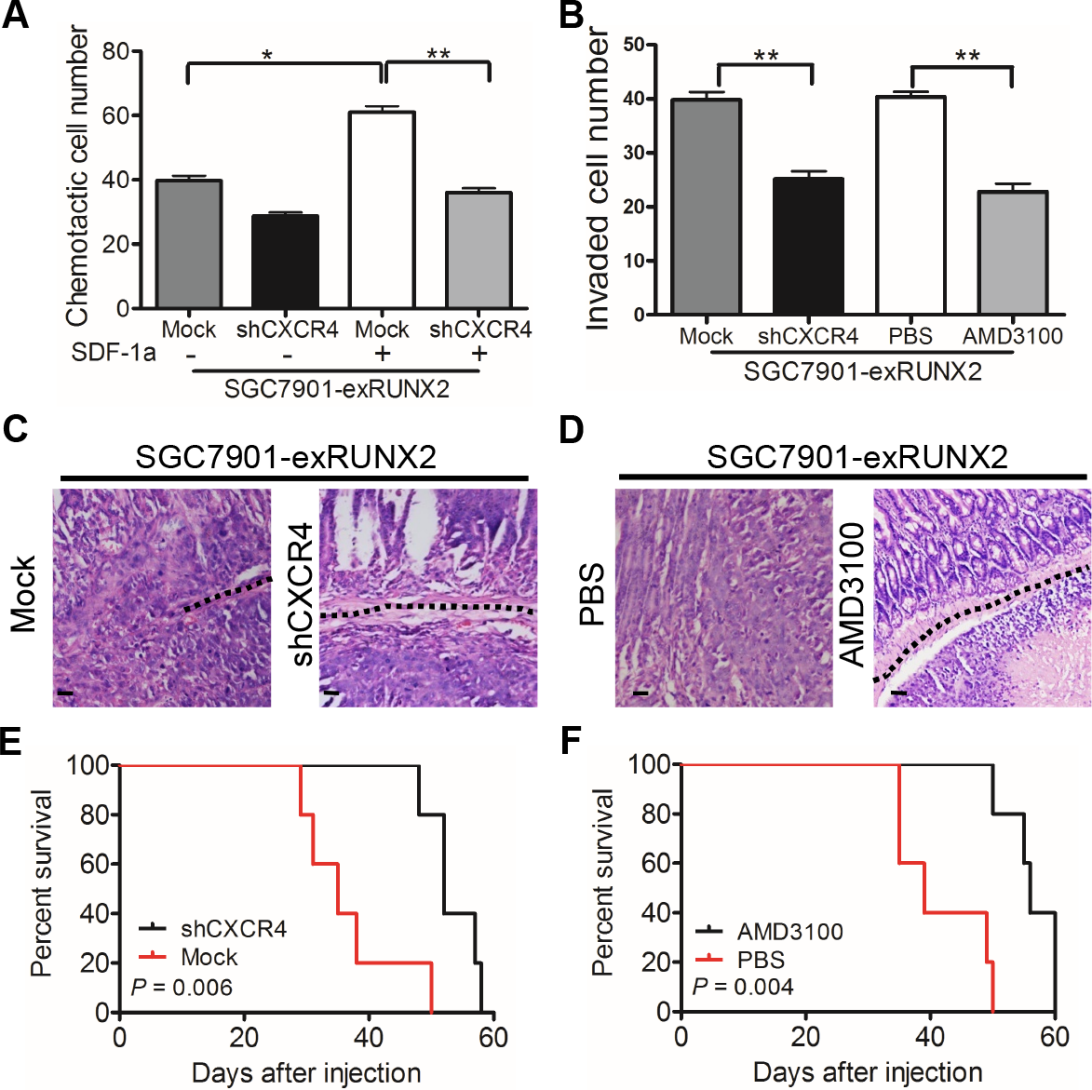
CXCR4 mediates RUNX2-promoted invasiveness and metastasis of GC cells (**A**) Chemotaxis of GC cells in response to the CXCR4 ligand SDF-1a (CXCL12, 10 nM/L). (**B**) Silencing CXCR4 or treatment with AMD3100 (50 ng/mL) attenuates the invasion ability of RUNX2-overexpressing SGC7901 cells. (**C**) Representative images of xenograft tumor section show that tumors formed by SGC7901-exRUNX2-Mock cells, but not SGC7901-exRUNX2-shCXCR4 cells, invade into the submucosa (black dotted line). (**D**) Representative images show that AMD3100 treatment reduced the invasion ability of RUNX2-overexpressing SGC7901 cells (black dotted line). Mice (*n* = 5 for each group) were intra-peritoneally injected with AMD3100 (7.5 mg/kg) every 3 days and the tumors were harvested after GC cell orthotopic implantation for 8 weeks. Scale bar *=* 50 μm. (**E** and **F**) Overall survival curves show that mice bearing tumors formed by SGC7901-exRUNX2 cells with CXCR4 knockdown or AMD3100 treatment have a long survival than mice implanted with mock or PBS treated SGC7901-exRUNX2 cells (*n* = 5 for each group). **P < 0.01, Student's *t* test.

Since CXCR4 has been reported to mediate tumor cell invasion and metastasis, we performed chemotaxis assays to examine the capacity of GC cells to migrate in response to SDF-1α (CXCL12), a specific ligand for CXCR4. SGC7901-exRUNX2 cells showed increased migration to SDF-1α, which was attenuated by CXCR4 silencing (SGC7901-exRUNX2-shCXCR4 cells, Supplementary Figure S1D, S1E) (Figure 6A and Supplementary Figure S3A). CXCR4 knockdown or treatment with the CXCR4 antagonist AMD3100 inhibited the invasiveness of RUNX2-overexpressing GC cells (Figure 6B and Supplementary Figure S3B). Upon orthotopic implantation, more tumors derived from SGC7901-exRUNX2-Mock cells implanted in the serosa invaded muscularis and submucosa regions of the mouse stomach than SGC7901-exRUNX2-shCXCR4 cells (*P* < 0.01; Figure 6C and Supplementary Table S5). Treatment of mice with the CXCR4 antagonist AMD3100 also attenuated the invasiveness of SGC7901-exRUNX2 cells (*P* < 0.05; Figure 6D and Supplementary Table S5). In addition, both CXCR4 knockdown or AMD3100 treatment markedly reduced liver metastasis of SGC7901-exRUNX2 cells (*P* < 0.05; Supplementary Table S5), in association with improved survival of tumor bearing mice (*P* < 0.01, Figure 6E, 6F). These results demonstrate that CXCR4 is a target gene of RUNX2 to promote the invasion and metastasis of GC cells.

## DISCUSSION

In this study, we demonstrated for the first time that the transcription factor RUNX2 was involved in the invasion and metastasis of human GC by up-regulating the expression of the chemokine receptor CXCR4. RUNX2 was also identified as an independent prognostic indicator for GC patients.

RUNX members are transcription factors involved in a variety of important pathophysiological processes [10–13]. In mammals, RUNX family consists of three members, RUNX1, RUNX2 and RUNX3, encoded by different genes but have a common Runt domain with 128 amino acids. The RUNX family controls a wide range of developmental processes with lineage and stage specificity [10–13]. Mice deficient in individual *Runx* genes displayed diverse phenotype, indicating the non-redundant, tissue-specific role of RUNX [10]. *Runx1* knockout mice showed disregulation of hematopoiesis; *Runx2* deficiency resulted in a complete lack of ossification and *Runx3* deletion led to gastrointestinal and neural disorders [14–18]. Recently, the RUNX family was also linked to cancer progression [13, 19]. RUNX1 and RUNX3 were originally considered as tumor suppressors [20, 21]. For instance, recurrent mutation of RUNX1 was observed in breast cancer, suggesting absence of RUNX1 may promote breast cancer development [22, 23]. Studies also showed that hypermethylation and subsequent silencing of RUNX3 are prevalent in solid tumors of the breast and stomach, suggesting a tumor suppressing role of RUNX3 [15, 20].

The role of RUNX2 in carcinogenesis and cancer progression may be tissue and context-dependent [10]. RUNX2 was found to promote early breast cancer progression [23]. Transcriptome profile showed that RUNX2 was associated with the malignant behavior of prostate cancer, including invasion and bone spread by up-regulating MMP9, SNAI2 and Smad3 [24]. In colon cancer, RUNX2 was suggested as an unfavorable prognostic factor that promotes the proliferation and invasion of tumor cells through an estrogen signal pathway [25]. Therefore, RUNX2 was considered as a potential oncogene. However, genome-wide expression profiling of breast cancer showed that RUNX2 attenuates the effect of estradiol on gene expression and colony formation by tumor cells, implying that RUNX2 may possess tumor suppressor properties in breast cancer [26]. Nevertheless, our present study demonstrates RUNX2 as an oncogene in GC, whose expression was positively correlated with the level of invasion and metastasis of GC in association with poor survival of patients.

The molecular basis for RUNX2 as an oncogene in cancer has been studied in several malignant tumors. The metastasis of melanoma to bone was likely caused by high concentrations of TGF-beta and activation of its target genes, including RUNX2 [27]. The expression of RUNX2 and androgen receptor (AR) predicts prostate cancer recurrence [28]. These observations are consistent with our present study in which the expression of RUNX2 is clearly correlated with the invasive and metastatic potential of human GC. However, RUNX2 was reported as only weakly expressed in GC tissue [29] and has tumor suppressor activity in GCs [30]. Some epigenetic modulation might explain this discrepancy [10]. RUNX3, another important member of the RUNX family, could suppress the TEAD-YAP oncogenic complex in gastric carcinogenesis [31]. YAP, which was a downstream factor of Hippo signaling, interacts with RUNX2 to play an oncogenic role in liver cancer [32]. Our data revealed that in human GC specimens, RUNX2 and RUNX3 levels were negatively correlated (Supplementary Figure S5), which might suggest that RUNX3 may contact the activity of RUNX2 in normal gastric tissues.

Our study demonstrated that the tumor-promoting activity of RUNX2 was attributable to its transcriptional up-regulation of the chemokine receptor CXCR4, which has been shown to mediate the invasion and metastasis of a variety of cancers [33, 34]. In human breast cancer, activation of CXCL12/CXCR4 pathway induces the chemotaxis, invasion and metastasis of tumor cells [35]. Activation of CXCR4 also promotes the growth and production of angiogenetic factors by glioma stem cells [36]. A subpopulation of migrating CD133^+^CXCR4^+^ cancer stem cells contributed to pancreatic cancer metastasis [37]. CXCR4 was additionally shown to be involved in early stage GC development through recruitment of stromal cells and establishment of progenitor niche to favor tumor growth [38]. In human colorectal cancer, the expression of CXCR4 was found correlated with the recurrence and liver metastasis [39]. However, how CXCR4 was up-regulated in tumor cells is an issue of debate. In GC specimens of our study, CXCR4 staining was found positively correlated with RUNX2 expression (Supplementary Figure S4). In addition, we found that CXCR4 in GC cells was up-regulated by direct binding of RUNX2 to the promoter region of CXCR4 gene to initiate its transcription. This reveals a mechanistic basis for CXCR4 over-expression in more highly malignant GC cells. Thus, disruption of RUNX2/CXCR4 axis may facilitate the development of anti-GC therapy. Researcher Fujita observed that the CXCR4 ligand SDF-1a was expressed in the peritoneal mesothelium [40]. While, our result indicated that SDF-1a was widely detectable in the cancerous and adjacent tissues of human GC (data not shown). It should be pointed out that RUNX2 may also control ligand-independent GC cell mobility as shown in the wound scratching assay. Without adding a chemoattractant RUNX2 also controls GC cell spreading into the wound area. Knockdown of naturally expressed RUNX2 in MGC803 cells also reduced cell migration response to SDF-1a (Supplementary Figure S6). Further study is required to delineate the multifacet effect of RUNX2 on GC malignancy.

In this study, we used an orthotopic mouse implantation model to study the role of RUNX2 and CXCR4 in GC invasion and metastasis *in vivo*. By definition, a true GC orthotopic model should initiate cancer growth from the intraluminal mucosal surface that progresses to deeper layers of the stomach wall [41]. But such an approach has shown technical limitations [41]. As an alternative, cancer cells are injected into the subserosa of the stomach wall for observation of a reversed invasion path [42–44]. In our study, we used matrigel to reduce the leakage of injected GC cells and false metastasis. This enables us to clearly show the relevance of RUNX2 and CXCR4 to GC invasion and metastasis *in vivo*.

The development and progression of GC are complex that involve a multitude of genetic and epigenetic changes culminating in the malignant transformation of normal epithelial cells. Our study indicates RUNX2 as an important oncogene and CXCR4 as an effector for GC invasion and metastasis, thus providing a significant progress in understanding the molecular basis for the progression of GC. Our results also demonstrate RUNX2 as a novel prognostic indicator and RUNX2/CXCR4 axis as a potential therapeutic target for GC.

## MATERIALS AND METHODS

### Patients and tumor samples

A total of 305 human GC with paired peritumoral gastric tissues were utilized in this study, including 199 patients who underwent curative resection between 2006 and 2007 at the Southwest Hospital (Third Military Medical University, Chongqing, China) and a tissue microarray (TMA) of 106 GC specimens collected between 2002 and 2005 (Shanghai Biochip Biotechnology, Shanghai, China). None of the patients received preoperative radiotherapy or chemotherapy. The study was performed with the consent of patients and approved by the Institutional Review Board for human study.

### Cell lines and primary GC cells

SGC7901 and MGC803 human GC cell lines were purchased from the Cell Bank of Shanghai Institute of Cell Biology, Chinese Academy of Sciences. Primary gastric cancer cell XN0422 (diffuse-type by Lauren classification) was described previously [45]. All the cells were grown in RPMI 1640 (Gibco, USA) supplemented with 10% fetal bovine serum (FBS, Gibco), 100 U/mL penicillin and 100 μg/mL streptomycin at 37°C in 5% CO_2_ moist air.

### Immunohistochemistry (IHC) and immunoreactivity scores (IRS)

Consecutive human GC tissue sections (4 μm) were made and mounted on silanized slides. The whole process was performed following the protocol of DAKO REAL EnVision Detection System (DAKO, Denmark). Primary mouse anti-RUNX2 antibody (1:200) was purchased from Sigma-Aldrich.

A semi-quantitative method was applied to evaluate immunoreactivity scores (IRS) of RUNX2 staining by multiplying staining intensity and the percentage of positive cells [46, 47]. The staining intensity was scored from 0–3 (0, negative; 1, weak; 2, moderate; and 3, strong). The percentage of positive cells was scored as 1 (under 25%), 2 (26–50%), 3 (51–75%) and 4 (76–100%). The best predictive IRS cut-off value was analyzed with X-tile and the cut-off value was determined as 3. IRS ≤ 3 was defined as low and IRS ≥ 4 was defined as high for expression of RUNX2. All slides were evaluated independently by two pathologists without the knowledge of the source of the samples. Interobserver and intraobserver variability was minimal.

### Gene transfection

For RUNX2 over-expression in GC cells, full length human RUNX2 was generated and inserted into a lentivirus vector. Lentiviral particles containing RUNX2 were packaged and named as Lentivirus-exRUNX2 (exRUNX2). Lentiviral particles packaged with a blank vector were used as a negative control (Control). ExRUNX2 and Control were transfected into RUNX2 negative SGC7901 cells with polybrene. Stably transfected SGC7901 cells were selected with a red fluorescence protein (RFP) marker. The over-expression of RUNX2 was verified by qPCR and Western blot and the cells were named as SGC7901-exRUNX2 or SGC7901-Control cells.

For RUNX2 knockdown from GC cells, three pairs of self-complementary hairpin DNA fragments targeting RUNX2 mRNA and control DNA were synthesized and cloned into a pMAGic 7.1 lentiviral vector. The packaged lentiviral particles containing shRUNX2 and control shRNA were named as Lentivirus-shRUNX2 (shRUNX2#1, #2 and #3) and Lentivirus-Mock (Mock), respectively (Supplementary Table S6). MGC803 cells or XN0422 cells expressing RUNX2 were infected with shRUNX2 or Mock at MOI of 10 in the presence of 6 μg/mL polybrene. Stably transfected MGC803 cells and XN0422 cells were selected with green fluorescence protein (GFP) and the efficiency of RUNX2 knockdown was verified by qPCR and WB.

In SGC7901-exRUNX2 cells, the overexpressed chemokine receptor CXCR4 was silenced with lentivirus-shCXCR4. The expression of CXCR4 was verified by qPCR and WB. The cells were named SGC7901-exRUNX2-shCXCR4.

### RNA extraction and quantitative PCR

Total RNA from SGC7901 and MGC803 GC cells was isolated using Trizol (Takara, Japan) according to the manufacturer's instructions. The RNA was reverse transcribed to cDNA using RevertAid^™^ First Strand cDNA Synthesis Kit (Fermentas, Canada) and amplified with PCR amplification primers in DreamTaq^™^ Green PCR Master Mix (Fermentas) on a CFX96 system (BioRad, USA). The method to calculate the primer efficiency was determined using a fixed single template concentration and serial dilutions of cDNA as described in Schmittgen's paper [48]. Primer sequences used in the experiments were listed in Supplementary Table S7.

### Western blot

GC cells were washed twice with ice-cold PBS and lysed with protein extraction reagent (Pierce, USA) containing 1% protease inhibitors (Pierce). Lysate was centrifuged at 4°C at 14,000 × g for 20 min and protein concentration in the supernatant was determined using the BCA protein assay kit (Pierce). Total protein was separated by 10% SDS-polyacrylamide gel electrophoresis and transferred to PVDF membranes (Millipore, USA), which were treated for 2 h at room temperature (RT) in PBS containing 5% bovine serum albumin (Sigma-Aldrich, USA) and 0.1% Tween-20. The membranes were then incubated at 4°C overnight with the following primary antibodies: mouse anti-RUNX2 (Sigma-Aldrich), rabbit anti-GAPDH (Cell Signaling Technology, USA), or mouse anti-CXCR4 (Abnova, Taiwan), followed by washing 3 times with PBST and 1 h incubation with a peroxidase conjugated secondary antibody (Byotime, China). Chemiluminescence was detected using SuperSignal West Femto Maximum Sensitivity Substrate (ECL, Pierce) in a ChemiDocXRS system (BioRad).

### Chromatin immunoprecipitation (ChIP)

Tumor cells grown in dishes were washed with PBS. Formaldehyde was added drop-wise to the dishes to a final concentration of 0.75% for cross-linking the proteins and DNA at RT for 10 min. Glycine was added to a final concentration of 125 mM and the dishes were shaken at RT for 5 min. The cells were then harvested in cold PBS. After centrifugation for 5 min, the supernatant was discarded and the cell pellets were resuspended in lysis buffer. After sonication, lysate was centrifuged and the supernatant was immunoprecipitated using a mouse anti-RUNX2 antibody (Cell Signaling Technology, USA) or a rabbit polyclonal antibody (Cell Signaling Technology) overnight at 4°C. Five pairs of primers used for qPCR analysis were listed in Supplementary Table S4.

### Luciferase reporter assay

Wild-type CXCR4 promoter (CXCR4-WT) and its mutant segments were cloned and ligated into a pGL3 vector. SGC7901-exRUNX2 cells were cultured in 24 well plates and grew to 80% confluence. Vectors containing CXCR4-WT and mutant CXCR4 promoters were then transfected into the cells together with a *Renilla* vector (Byotime, China). After 24 h, the cells were harvested and the luciferase activity of *firefly* and *Renilla* was determined using a Dual-Luciferase Reporter Assay system (Promega, USA). The results were normalized against *firefly* luciferase activity as a control.

### Electrophoretic mobility shift assay (EMSA)

The probes, CGGAGTGGTTTGACC for WT CXCR4 promoter (WT) and CGGGAGAACTTGACC for CXCR4 mutant promoter (Mut), were labeled with ^32^P. Nuclear extracts were prepared from SGC7901-exRUNX2 cells. The reaction mixtures of DNA-protein complex were prepared as follows: nuclear extracts (18 μg) and labeled probe (WT or Mut CXCR4 promoter, 1 ng) for specific nuclear extract binding reaction; labeled probe without nuclear extracts for negative reaction; mixture of nuclear extracts, labeled probe (1 ng) and unlabeled probe (300 ng) for cold competition reaction; mixture of nuclear extracts, labeled probes (WT or Mut CXCR4 promoter) and IgG (200 ng) or anti-RUNX2 antibody (200 ng) for supershift reaction. All reaction mixtures were prepared with DNA binding buffer containing poly (dI-dC). The DNA-protein complexes were resolved on 4% non-denaturing polyacrylamide gels for 2 h. The gel was dried for 1 h at 65°C and then exposed to an X-ray film (Kodak, USA) for autoradiography.

### *In vitro* cell invasion and cell scratching assays

To test the invasiveness of GC cells, Transwell chambers (8 μm pore size, Millipore) were coated with 10 μL of 1:1 (v/v) RPMI-1640-diluted matrigel (Growth Factor Reduced Matrigel Matrix, BD, USA). Cell suspension was placed into the upper chambers at the density of 5 × 10^4^ cells in 200 μL serum-free RPMI-1640. The lower chambers were filled with 600 μL RPMI-1640 containing 10% FBS. After incubation for 24 h at 37°C, the cells were fixed with 4% formaldehyde for 20 min. Cells on the upper surface of the filter membranes were scraped with a cotton swap, and the cells on the lower surface of the membranes were stained with 1% crystal violet and counted in five high-power fields under light microscopy. For AMD3100 treatment, GC cells were pre-incubated with AMD3100 (50 ng/mL) for 6 h before the invasion assay.

For cell scratching assay, GC cells were cultured in 6 cm dishes to 90% confluence. Wounds were created by scratching the cell monolayer with 10 μL peptide tips. Cells crossing the scratched lines were monitored at 0 h and 24 h. Pictures of scratched areas were taken and measured. Percentage of cells moving into the scratched area was counted for further analysis.

### Chemotaxis assay

Chemotaxis assay was performed with transwell chambers (8 μm pore size, Millipore) without matrigel coating. The upper wells of the chamber were added with 5 × 10^4^ cells suspended in 200 μL serum-free medium. Lower wells of the chamber were added with 600 μL serum-free medium containing 10 nM/L SDF-1α (CXCL12, Sigma-Aldrich). After an incubation period of 6 h at 37°C, migrated cells on the lower surface of membrane were counted in five randomly chosen fields.

### Orthotopic implantation of GC cells in nude mice

Five-week-old male BALB/C-nu mice were purchased from the Laboratory Animal Center of Southwest Hospital (Chongqing, China). A modified mouse orthotopic GC implantation model was established based on previously reports [49, 50]. Briefly, nude mice were fasted for 12 h and deprived of water for 4 h before abdominal operation. Lidocaine hydrochloride (6 μL/g) was intraperitoneally injected for anesthesia. GC cells were suspended in a mixture of matrigel^™^ (BD, USA) and PBS (v/v, 1:2) at a concentration of 1 × 10^7^ cells/mL and 50 μL suspensions were injected into the subserosa of mouse stomach with a 29 G needle (BD, USA). Mice were intra-peritoneally injected with AMD3100 (7.5 mg/kg) every 3 days as a treatment group. Eight weeks after GC cell implantation, mouse stomach, liver and peritoneal cavity were carefully examined for invasion and metastasis. The organs were sectioned at 5 μm for Gill's H & E staining. The survival of the animals with orthotopic GC cell implantation was also recorded. Animal experiments were carried out following the guidelines of Humane Care and Use of Laboratory Animals and approved by the Institutional Animal Care and Use Committee of the Hospital.

### Statistical analysis

All experiments were performed at least 3 times and results from representative experiments are presented. The mean ± SD values were analyzed by Student’ s *t* test. Statistical analysis was performed using SPSS20.0 software (IBM, USA) and GraphPad Prism. The cut-off value of score for RUNX2 IHC staining was analyzed with X-tile. Chi-square analysis was used to evaluate the relationship between RUNX2 positive rate and the clinicopathological features of GC specimens. The overall survival (OS) of GC patients was calculated with Kaplan-Meier method. COX's proportional hazard regression model was established for multivariate analysis of the combinational contribution of RUNX2 and clinicopathological features to the OS of patients. *P* < 0.05 was considered as statistically significant.

## SUPPLEMENTARY MATERIALS FIGURES AND TABLES


