# Supplementary material for: Transcription factor RUNX2 up-regulates chemokine receptor CXCR4 to promote invasive and metastatic potentials of human gastric cancer
Source: Oncotarget. 2016 Mar 21;7(15):20999–1012. doi: 10.18632/oncotarget.8236 (PMC4991507; doi:10.18632/oncotarget.8236)
Supplement: Supplementary file 1 [file oncotarget-07-20999-s001.pdf]

Transcription factor RUNX2 up-regulates chemokine receptor CXCR4 to promote invasive and metastatic potentials of human gastric cancer

Supplementary Materials

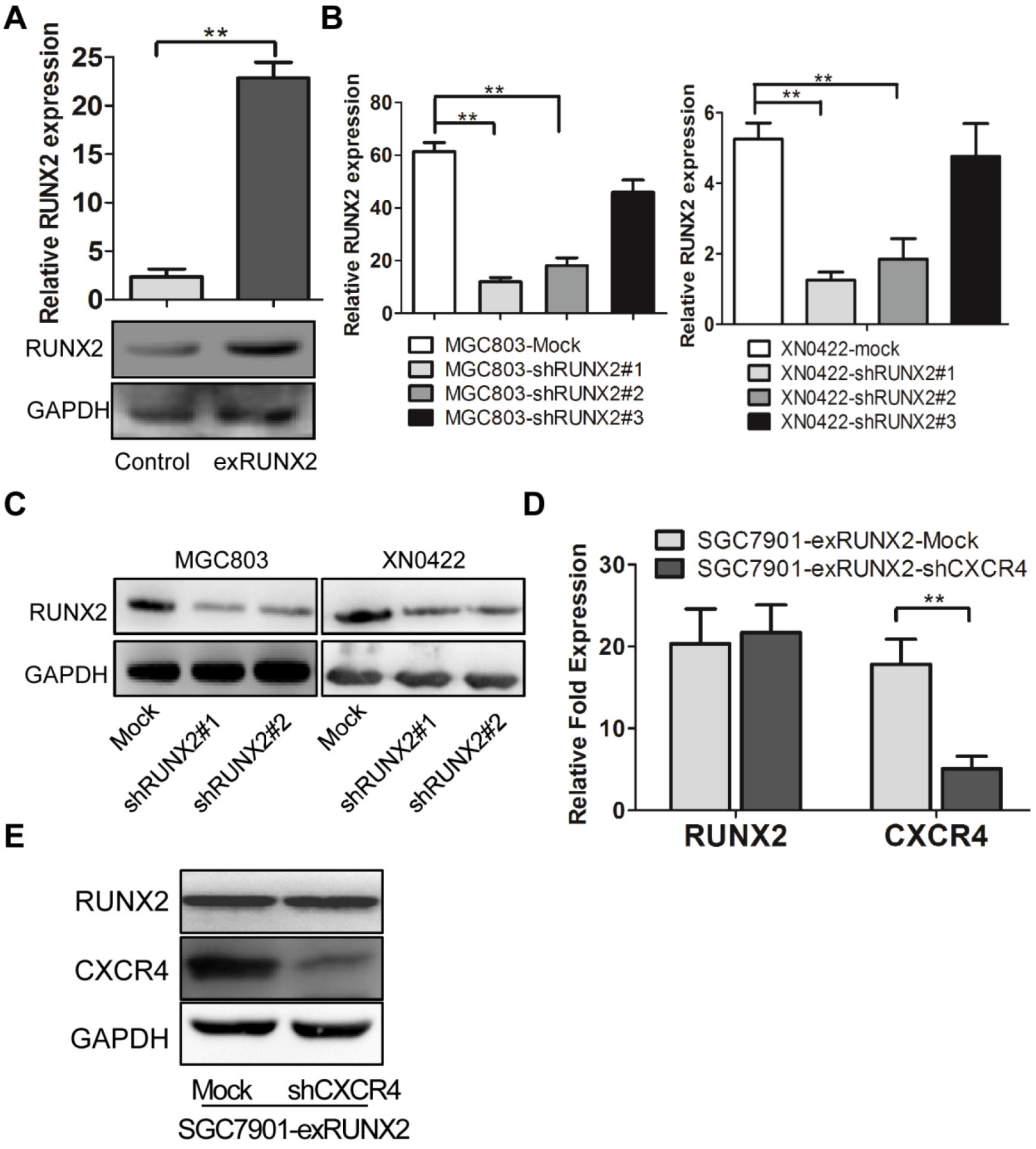

Supplementary Figure S1: The efficiency of gene transfection. (**A**) RUNX2 is significantly up-regulated in RUNX2-overexpressing SGC7901 cells as examined by qPCR and WB. (**B** and **C**) RUNX2 is significantly silenced with shRNA in MGC803 and XN0422 cells as examined by qPCR and WB. (**D** and **E**) qPCR and WB show the efficiency of CXCR4 knockdown in RUNX2-overexpressing SGC7901 cells. All results of qPCR are presented as relative fold expression normalized with the level of housekeeping gene glyceraldehyde-3-phosphate dehydrogenase (GAPDH). *$P < 0.05$, **$P < 0.01$, Student's *t* test.

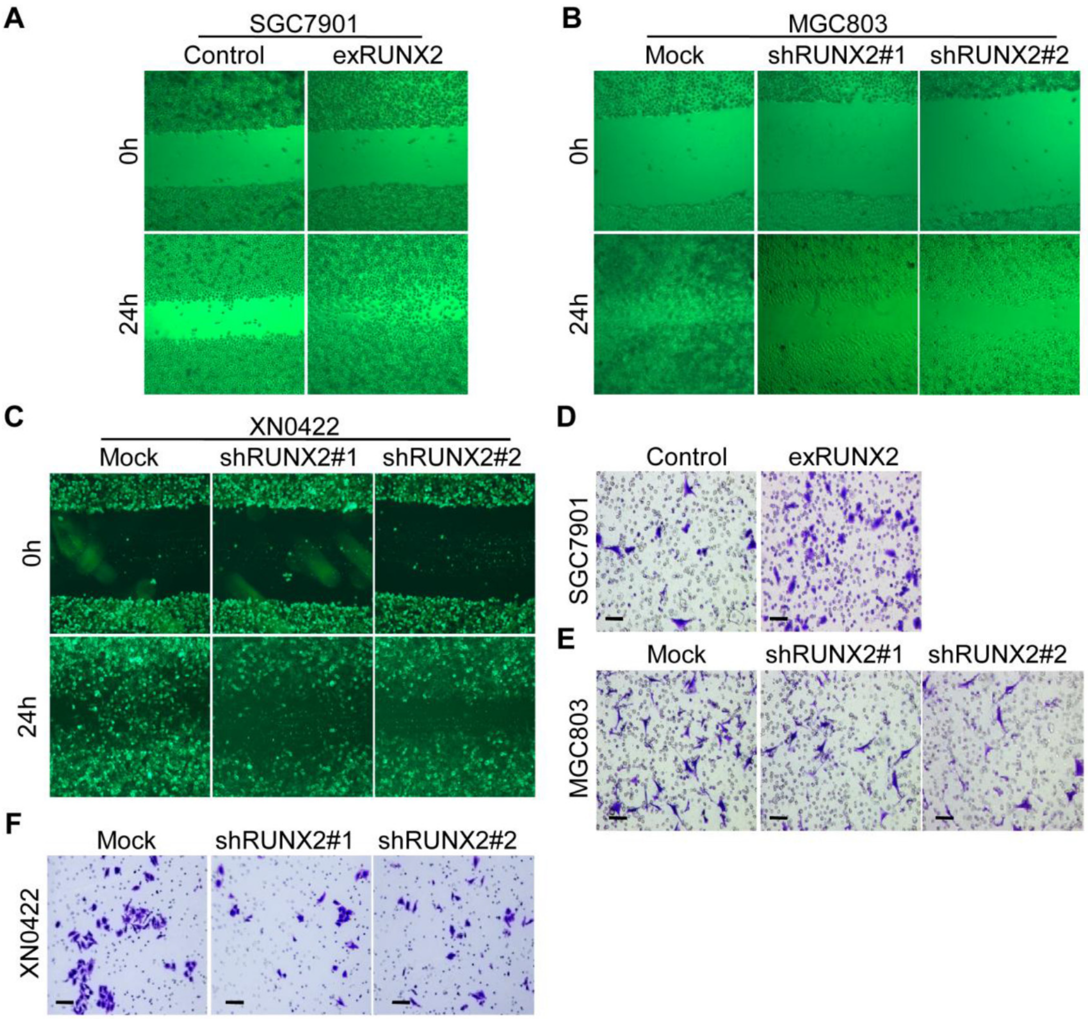

Supplementary Figure S2: Representative images for the effect of RUNX2 on the migration and invasion abilities of GC cells. (**A**) Cell scratching assay of SGC7901-exRUNX2 and control cells. (**B** and **C**) Cell scratching assays of MGC803 and XN0422 cells transfected with shRUNX2 constructs (shRUNX2#1 and #2) or mock vector. (**D**) Cell invasion assay for SGC7901-exRUNX2 and control cells. (**E**) The invasion assay of MGC803-mock and MGC803-shRUNX2#1 and #2 cells. (**F**) The invasion assay of XN0422-mock and XN0422-shRUNX2#1 and #2 cells. Scale bar = 50 μm.

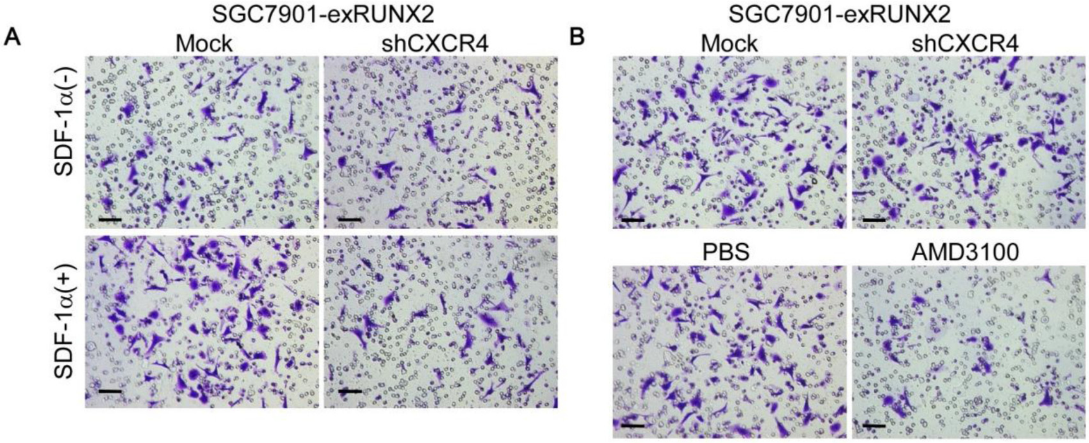

Supplementary Figure S3: Representative images of chemotaxis and invasion of GC cells mediated by CXCR4. (**A**) Chemotaxis of GC cells in response to the CXCR4 ligand SDF-1α (CXCL12, 10 nM/L). (**B**) Inhibition of SDF-1α-induced invasion of SGC7901-exRUNX2 cells by CXCR4 knockdown or AMD3100 treatment (50 ng/mL). Scale bar = 50 μm.

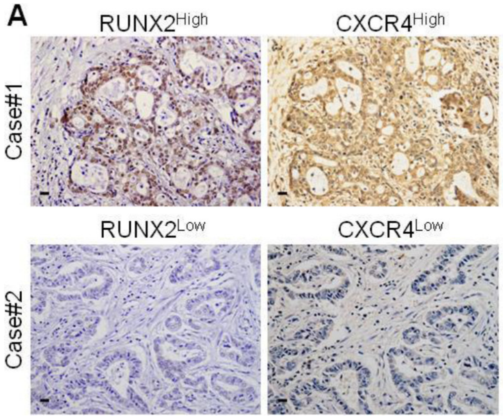

B

Table. Correlation between RUNX2 and CXCR4 IHC staining in GC tissues.

	CXCR4		P value
RUNX2	Low	High	
Low	40	45	<0.001
High	46	174	

Supplementary Figure S4: RUNX2 and CXCR4 IHC staining in continuous GC sections. (**A**) Representative staining of CXCR4 and RUNX2 in human GC specimens. Scale bar = 50 μm. (**B**) The correlation between the expression of CXCR4 and RUNX2 in human GC specimens.

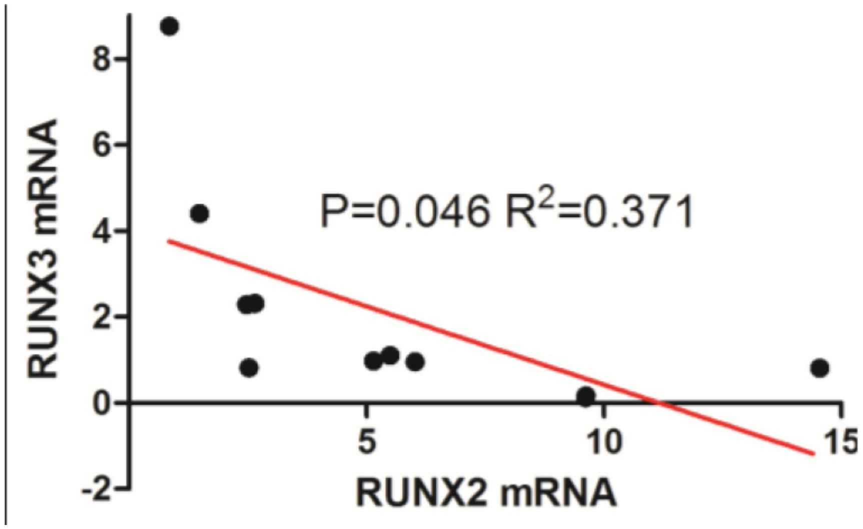

Supplementary Figure S5: Lineage regression analysis of RUNX2 and RUNX3 mRNA levels. p = 0.046, R^2 = 0.371

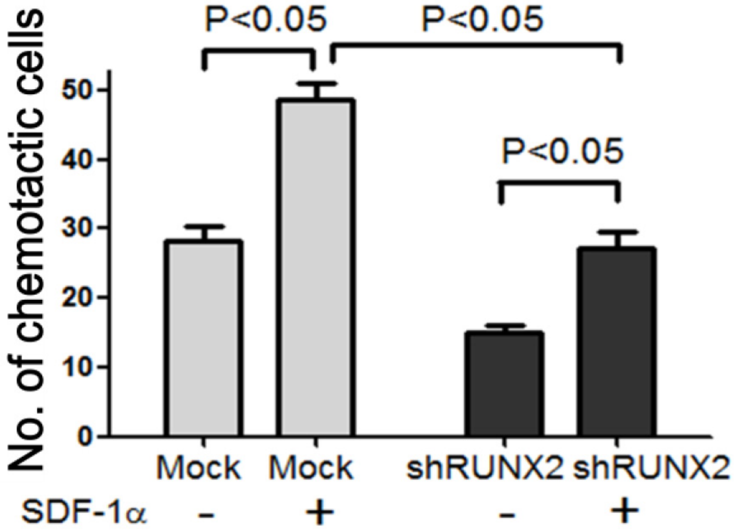

Supplementary Figure S6: Chemotaxis response of the GC cell MGC803 naturally expressing RUNX2 to SDF-1α (10 nM/L) with or without RUNX2 silencing. Silencing RUNX2 significantly reduced cell migration in response to SDF-1α ($P < 0.05$).

Supplementary Table S1: Univariate and multivariate analysis of RUNX2 expression and clinicopathological features for predicting the survival of human GC patients

Feature	Univariate analysis		Multivariate analysis	
	HR (95%CI)	*p* value	HR (95%CI)	*p* value
Age	1.009 (0.992–1.027)	0.305	1.000 (0.984–1.018)	0.965
Gender	0.822 (0.524–1.290)	0.394	0.837 (0.523–1.339)	0.458
TNM stage	1.179 (0.953–1.459)	0.130	1.293 (0.846–1.975)	0.235
Differentiation degree	1.227 (0.860–1.752)	0.260	0.704 (0.488–1.016)	0.061
Lymph node metastasis	2.520 (1.596–3.979)	< 0.000	1.539 (1.089–2.591)	0.045
Depth of invasion	2.587 (1.735–3.858)	< 0.000	1.620 (1.022–2.567)	0.040
RUNX2 expression	2.010 (1.119–3.612)	0.019	3.454 (1.408–8.230)	0.007

Supplementary Table S2: Influence of RUNX2-overexpression or -knockdown on the invasion and metastasis of GC xenograft in nude mice

		Mucosa invasion				Liver metastasis			
	**Cells**	**Ex#. 1**	**Ex. 2**	**Ex. 3**	***P* value**	**Ex. 1**	**Ex. 2**	**Ex. 3**	***P* value**
SGC7901	Control	0/5	1/5	2/5	0.016	0/5	1/5	0/5	0.047
	exRUNX2	4/5	4/5	3/5		2/5	2/5	1/5	
MGC803	Mock	5/5	4/5	5/5	0.001	4/5	4/5	3/5	0.008
	shRUNX2	0/5	1/5	1/5		1/5	2/5	1/5	
XN0422	Mock	4/5	4/5	5/5	0.003	3/5	3/5	4/5	0.013
	shRUNX2	1/5	1/5	2/5		1/5	1/5	2/5	

Note: Ex# Experiment.

Supplementary Table S3: Predicted invasion-related candidate RUNX2-targetting genes by bioinformatic analysis

Genes	Scores	Strand	Predicted site sequences
MMP7	8.254	1	TTTTTTTGTGTTTAA
MMP9	7.129	1	CTGGAGTGTGGGGAG
	7.028	1	AGGGCCTGCGGTTTC
	8.346	1	CGGGTCTGGGGTCTT
MMP13	6.946	1	TCTCTCTGTAGTTAT
CXCR4	11.462	1	CGGAGTGGTTTGACC
E-Cadherin	8.913	1	TGGCGTGGTGGTGTG
	9.056	1	ATTGGCTGTGGCCGG
N-Cadherin	9.071	1	GCTCTTTGTGGGTGC

Supplementary Table S4: Sequences of primers for chromatin-immunoprecipitation (ChIP)

Primer Sequences	Product length (bp)	Product sites
1F: TGGCATTTCATCTCTCCGGG<br>1R: CGCCTAGAACAGTGCGTGGC	96	–2875 to –2780
2F: CAGCGGTTACCATGGAGGG<br>2R: CGTTTGAACTTAGAGCGCAG	86	–1847 to –1762
3F: GCAGTTCGAGAGTTTGGGGT<br>3R: CCCTGAGATTTGCGCTCCGG	96	–1081 to –986
4F: GTTTGGCTCTCTCCGAGTCC<br>4R: GAAACCCTCTTGCTAGGGAG	81	–885 to –805
5F: GGAGTTAGCCAAGATGTGAC<br>5R: GAGGAGAGTTGTAGGATTC	143	13 to 155

Supplementary Table S5: The effect of CXCR4 knockdown and AMD3100 treatment on the invasion and metastasis of GC xenograft tumors in nude mice

SGC7901	Mucosa invasion			*P* value	Liver metastasis			*P* value
	Ex#. 1	Ex. 2	Ex. 3		Ex. 1	Ex. 2	Ex. 3	
exRUNX2-mock	4/5	4/5	5/5	0.003	2/5	2/5	3/5	0.024
exRUNX2-shCXCR4	1/5	1/5	2/5		1/5	0/5	1/5	
exRUNX2 +PBS	5/5	5/5	3/5	0.023	2/5	3/5	2/5	0.013
exRUNX2+AMD3100	1/5	2/5	2/5		0/5	1/5	0/5	

Note: Ex#, Experiment.

Supplementary Table S6: Sequences of RUNX2 shRNA and Mock RNA

shRNA	Sequence (5′ - 3′)
#1	CCGGGTGGTCCTATGACCAGTCTCGGGATCCAAAGACTGGTCATA GGACCACTTTTTG
#2	CCGGCCTTGACCATAACCGTCTTCTCAAGAGAAAGACGGTTATGGT CAAGGTTTTTG
#3	CCGGGCACTCCATATCTCTACTATTCAAGAGATAGTAGAGATATGGA GTGCTTTTTG
Mock	CCGGTTCTCCGAACGTGTCACGTTTCAAGAGAACGTGACACGTTCG GAGAATTTTG

Supplementary Table S7: Sequences of primers for real-time PCR

**Name**	**Sequence**
RUNX2	F: 5′-GTTTGTTCTCTGACCGCCTC-3′<br>R: 5′-CCAGTTCTGAAGCACCTGA-3′
CXCR4‡	F: 5′-ACTACACCGAGGAAATGGGCT-3′<br>R: 5′-CCCACAATGCCAGTTAAGAAGA-3′
GAPDH	F: 5′-AAGGTGAAGGTCGGAGTCAAC-3′<br>R: 5′-GGGGTCATTGATGGCAACAATA-3′
MMP7	F: 5′-GATTGGCTTTGCGCGAGGAGC-3′<br>R: 5′-TACCATCCGTCCAGCGTTCATCCT-3′
MMP9	F: 5′-GCGGAGCACGGAGACGGGTA-3′<br>R: 5′-CCGAGTTGGAACCACGACGCC-3′
MMP13	F: 5′-GCCTGCTGGCTCATGCTTTTCCT-3′<br>R: 5′-GGGTCCTTGGAGTGGTCAAGACCT-3′
E-Cadherin‡	F: 5′-CCCACCACGTACAAGGGTC-3′<br>R: 5′-ATGCCATCGTTGTTCACTGGA-3′
N-Cadherin‡	F: 5′-TTTGATGGAGGTCTCCTAACACC-3′<br>R: 5′-ACGTTTAACACGTTGGAAATGTG-3′

‡ primer sequences were selected from primer bank.
